# Supplementary material for: Severity of COVID-19 among Hospitalized Patients: Omicron Remains a Severe Threat for Immunocompromised Hosts
Source: Viruses. 2022 Dec 8;14(12):2736. doi: 10.3390/v14122736 (PMC9783877; doi:10.3390/v14122736)
Supplement: Supplementary file 1 [file viruses-14-02736-s001.zip › viruses-2000570-supplementary.pdf]

Title

Severity of COVID-19 among hospitalized patients: Omicron remains a severe threat for immunocompromised hosts

Authors:

Louis Nevejan[1,#], Sien Ombelet[1,2], Lies Laenen[1,2], Els Keyaerts[2], Thomas Demuyser[3,4], Lucie Seyler[5], Oriane Soetens[3], Els Van Nedervelde[5], Reinout Naesens[6], Dieter Geysels[6], Walter Verstrepen[6], Lien Cattoir[7], Steven Martens[7], Charlotte Michel[8], Elise Mathieu[8], Marijke Reynders[9], Anton Evenepoel[9], Jorn Hellemans[9], Merijn Vanhee[9], Koen Magerman[10], Justine Maes[10], Veerle Matheeussen[11], Hélène Boogaerts[11], Katrien Lagrou[1,2], Lize Cuypers[1,2,#,*] & Emmanuel André[1,2,#,*]

[#] corresponding authors - * shared last authorship

Louis Nevejan: - Lize Cuypers: lize.cuypers@uzleuven.be - Emmanuel Andre: Emmanuel.andre@uzleuven.be

Department of Laboratory Medicine, National Reference Center for Respiratory Pathogens, UZ Leuven –

University Hospitals Leuven

Herestraat 49, 3000 Leuven – BELGIUM

Supplemental Table & Figures

Supplemental Table S1. Clinical summary of newly hospitalized adult patients (≥18 years old) with positive SARS-CoV-2 screening result.

	All patients N = 1291	COVID-19 outcomes		
		Moderate/severe/ critical/fatal COVID-19 N = 538 (41.7%)	ICU admission due to COVID-19 N = 129 (10.0%)	In-hospital mortality related to COVID-19 N = 99 (7.7%)
**Patient characteristics**				
Age (years); median (IQR)	69 (50-83)	70 (59-82)	63 (53-72)	78 (68-86)
Male; n (%)	619 (47.9%)	310 (57.6%)	83 (64.3%)	63 (63.6%)
Immunocompromised; n (%)	286 (22.2%)	157 (29.2%)	51 (39.5%)	39 (39.4%)
**SARS-CoV-2 screening**				
Omicron variant, n (%)	905 (70.1%)	323 (60.0%)	68 (52.7%)	58 (58.6%)
Omicron BA.1 variant, n (%)	538 (41.7%)	294 (54.6%)	43 (33.3%)	31 (31.3%)
Omicron BA.2 variant, n (%)	34 (2.6%)	14 (2.6%)	0 (0.0%)	1 (1.0%)
Delta variant, n (%)	166 (12.9%)	126 (23.4%)	44 (34.1%)	31 (31.3%)
Untyped, n (%)	220 (17.0%)	89 (16.5%)	17 (13.2%)	10 (10.1%)
**Serology data at admission**				
IgG anti-N available, n (%)	117 (9.1%)	62 (11.5%)	20 (15.5%)	15 (15.2%)
Positive IgG anti-N; n (%)	15/117 (12.8%)	9/62 (28.1%)	2/20 (10.0%)	3/15 (20.0%)
IgG anti-S available, n (%)	194 (15.3%)	100 (18.6%)	36 (27.9%)	24 (24.2%)
Positive IgG anti-S; n (%)	142/194 (73.2%)	61/100 (61.0%)	19/36 (52.8%)	17/24 (70.8%)
**Hospitalization data**				
Hospital length-of-stay non-deceased patients (days); median (IQR)	8 (3-18)	8 (5-18)	22 (12-51)	
Hospital length-of-stay deceased patients (days); median (IQR)	20 (7-27)	13 (6-25)	19 (12-32)	13 (6-25)
ICU admission; n (%)	178 (13.8%)			
Deceased in hospital; n (%)	139 (10.8%)			
**Vaccination status**				
Vaccination status unknown; n (%)	120 (9.3%)	52 (9.7%)	6 (4.7%)	30 (30.3%)
Unvaccinated; n (%)	221 (17.2%)	112 (22.7%)	34 (26.4%)	14 (14.1%)
½ basic vaccination; n (%)	15 (1.2%)	6 (1.2%)	0 (0.0%)	1 (0.1%)
Basic vaccination; n (%)	263 (20.4%)	104 (19.3%)	34 (26.4%)	14 (14.1%)
Boosted; n (%)	672 (52.1%)	264 (49.1%)	55 (42.6%)	40 (40.4%)
Type of vaccines available; n (%)	892 (69.1%)	351 (80.1%)	82 (63.6%)	40 (40.4%)
Viral vector vaccinated; n (%)	262/892 (29.4%)	119/351 (33.9%)	32/82 (39.0%)	14/40 (35.0%)
Time since last vaccination available; n [%]	870 (67.4%)	337 (62.6%)	73 (56.6%)	31 (31.3%)
Vaccinated <2 months before admission; n [%]	215/870 (24.7%)	62/337 (18.4%)	13/73 (17.8%)	5/31 (16.1%)
Vaccinated <4 months before admission; n [%]	486/870 (55.9%)	218/337 (64.7%)	43/73 (58.9%)	23/31 (74.2%)
Vaccinated <6 months before admission; n [%]	739/870 (84.9%)	283/337 (84.0%)	61/73 (83.6%)	27/31 (87.1%)
**SARS-CoV-2 reinfection**				
Documented previous infection; n [IQR]	17 (1.3%)	4 (0.7%)	1 (0.8%)	1 (1.0%)

Supplemental Table S2. Clinical summary of newly hospitalized children (<18 years old) with positive SARS-CoV-2 screening result.

	All patients N = 210	COVID-19 outcomes		
		Moderate/severe/ critical/MIS-C COVID-19 N = 50 (23.8%)	ICU admission due to COVID-19 N = 8	In-hospital mortality related to COVID-19 N = 0 (0.0%)
**Patient characteristics**				
Age (years); median (IQR)	1 (1-6)	1 (1-5)	1 (1-1)	
Male; n (%)	128 (60.1%)	18 (36.0%)	6 (75.0%)	
Immunocompromised; n (%)	23 (10.1%)	4 (8.0%)	1 (12.5%)	
**SARS-CoV-2 screening**				
Omicron variant, n (%)	125 (59.5%)	22 (44.0%)	3 (37.5%)	
Omicron BA.1 variant, n (%)	83 (39.5%)	22 (44.0%)	2 (25.0%)	
Omicron BA.2 variant, n (%)	6 (2.9%)	0 (0.0%)	0 (0.0%)	
Delta variant, n (%)	21 (10.0%)	2 (4.0%)	1 (12.5%)	
Untyped, n (%)	58 (27.6%)	26 (52.0%)	4 (50.0%)	
**Serology data at admission**				
IgG anti-N available, n (%)	6 (2.9%)	2 (4.0%)	0 (0.0%)	
Positive IgG anti-N; n (%)	3/6 (50.0%)	2/2 (100.0%)		
IgG anti-S available, n (%)	4 (1.9%)	1 (2.0%)	0 (0.0%)	
Positive IgG anti-S; n (%)	4/4 (100.0%)	1/1 (100.0%)		
**Hospitalization data**				
Hospital length-of-stay non-deceased patients (days); median (IQR)	3 (2-5)	4.5 (2-7)	21 (10-35)	
ICU admission; n (%)	20 (9.5%)			
Deceased in hospital; n (%)	0 (0.0%)			
**Vaccination status**				
Vaccination status unknown; n (%)	12 (5.7%)	7 (14.0%)	3 (37.5%)	
Unvaccinated; n (%)	186 (88.6%)	43 (86.0%)	5 (62.5%)	
½ basic vaccination; n (%)	3 (1.4%)	0 (0.0%)		
Basic vaccination; n (%)	6 (2.9%)	0 (0.0%)		
Boosted; n (%)	3 (1.4%)	0 (0.0%)		
Type of vaccines available; n (%)	12 (5.7%)			
Viral vector vaccinated; n (%)	0/12 (0.0%)			
Time since last vaccination available; n [%]	12 (5.7%)			
Vaccinated <2 months before admission; n [%]	3/12 (25.0%)			
**SARS-CoV-2 reinfection**				
Documented previous infection; n [IQR]	0 (0.0%)			

Supplemental Table S3. Mixed-model logistic regression analysis in adult patients infected with Delta (n=166). Covariates: 1. odds increase per year; 2. versus female; 3. versus non-immunocompromised; 4. versus any vaccination; 5. versus unvaccinated or (1/2) basic vaccination; 6. versus vaccinated with mRNA solely. 7. versus unvaccinated or vaccinated >2/>4/>6 months before admission. Abbreviations: OR, odds ratio; CI; confidence interval.

	**Crude OR**	**95% CI**	**p value**	**Adjusted OR**	**95% CI**	**p value**
**Outcome 1** – disease severity: odds of moderate/severe/critical/fatal COVID-19 (n=126/166, 75.9%)						
Age at admission[1]	1.02	1.00-1.04	0.10	1.01	0.99-1.04	0.16
Male[2]	1.63	0.80-3.37	0.18	1.50	0.72-3.14	0.28
Immunocompromised[3]	1.12	0.52-2.51	0.77	1.01	0.45-2.32	0.97
Unvaccinated[4]	1.36	0.62-3.13	0.46	-	-	-
Boosted[5]	0.84	0.38-1.91	0.67	-	-	-
Viral vector vaccination[6]	1.93	0.67-6.43	0.25	-	-	-
Vaccinated <2 months before admission[7]	1.17	0.32-5.64	0.82	-	-	-
Vaccinated <4 months before admission[7]	0.48	0.16-1.34	0.18	-	-	-
Vaccinated <6 months before admission[7]	0.40	0.09-1.37	0.18	-	-	-
**Outcome 2** – ICU admission: odds of admission or transferred to ICU due to COVID-19 (n=44/166, 26.5%)						
Age at admission[1]	0.99	0.98-1.01	0.59	0.99	0.97-1.10	0.36
Male[2]	2.23	1.09-4.78	***0.03***	2.37	1.14-5.16	***0.02***
Immunocompromised[3]	0.99	0.47-2.06	0.99	1.05	0.42-2.31	0.91
Unvaccinated[4]	1.44	0.69-2.97	0.32	-	-	-
Boosted[5]	0.61	0.26-1.33	0.23	-	-	-
Viral vector vaccination[6]	2.53	0.91-7.02	0.07	-	-	-
Vaccinated <2 months before admission[7]	0.27	0.01-1.55	0.23	-	-	-
Vaccinated <4 months before admission[7]	0.44	0.15-1.25	0.12	-	-	-
Vaccinated <6 months before admission[7]	0.80	0.26-2.80	0.71	-	-	-
**Outcome 3** – mortality: odds of in-hospital mortality related to COVID-19 (n=31/166, 18.7%)						
Age at admission[1]	1.04	1.02-1.08	***0.002***	1.04	1.01-1.08	***0.005***
Male[2]	1.47	0.66-3.44	0.35	1.34	0.59-3.15	0.49
Immunocompromised[3]	2.46	1.08-5.78	***0.03***	2.12	0.92-4.87	0.07
Unvaccinated[4]	0.95	0.36-2.36	0.92	-	-	-
Boosted[5]	2.44	0.97-6.07	***0.05***	-	-	-
Viral vector vaccination[6]	1.53	0.36-5.85	0.54	-	-	-
Vaccinated <2 months before admission[7]	2.47	0.33-13.16	0.31	-	-	-
Vaccinated <4 months before admission[7]	4.30	0.69-83.33	0.19	-	-	-
Vaccinated <6 months before admission[7]	NA	NA	0.99	-	-	-

Supplemental Table S4. Mixed-model logistic regression analysis adult patients infected with Omicron (n=905). Covariates: 1. odds increase per year; 2. versus female; 3. versus non-immunocompromised; 4. versus any vaccination; 5. versus unvaccinated or (1/2) basic vaccination; 6. versus vaccinated with mRNA solely. 7. versus unvaccinated or vaccinated >2/>4/>6 months before admission. Abbreviations: OR, odds ratio; CI; confidence interval.

	**Crude OR**	**95% CI**	**p value**	**Adjusted OR**	**95% CI**	**p value**
**Outcome 1** – disease severity: odds of moderate/severe/critical/fatal COVID-19 (n = 323/905, 36%)						
Age at admission[1]	1.02	1.1-1.03	***<0.001***	1.02	1.01-1.03	***<0.001***
Male[2]	2.20	1.65-2.94	***<0.001***	2.02	1.51-2.73	***<0.001***
Immunocompromised[3]	2.18	1.55-3.07	***<0.001***	2.36	1.65-3.38	***<0.001***
Unvaccinated[4]	1.00	0.65-1.53	0.99	-	-	-
Boosted[5]	1.25	0.92-1.70	0.16	-	-	-
Viral vector vaccination[6]	1.30	0.93-1.83	0.12	-	-	-
Vaccinated <2 months before admission[7]	0.57	0.38-0.82	***0.003***	-	-	-
Vaccinated <4 months before admission[7]	1.01	0.71-1.42	0.98	-	-	-
Vaccinated <6 months before admission[7]	1.18	0.74-1.90	0.50	-	-	-
**Outcome 2** – ICU admission: odds of admission or transferred to ICU due to COVID-19 (n = 68/905, 7.5%)						
Age at admission[1]	0.99	0.98-1.00	0.08	0.99	0.98-1.00	0.07
Male[2]	1.76	1.07-2.95	***0.03***	1.72	1.03-2.92	***0.04***
Immunocompromised[3]	3.65	2.19-6.05	***<0.001***	3.41	2.04-5.68	***<0.001***
Unvaccinated[4]	0.99	0.42-2.03	0.98	-	-	-
Boosted[5]	0.94	0.56-1.64	0.83	-	-	-
Viral vector vaccination[6]	1.18	0.63-2.12	0.59	-	-	-
Vaccinated <2 months before admission[7]	0.97	0.48-1.86	0.94	-	-	-
Vaccinated <4 months before admission[7]	0.86	0.47-1.64	0.63	-	-	-
Vaccinated <6 months before admission[7]	0.93	0.43-2.32	0.86	-	-	-
**Outcome 3** –mortality: odds of in-hospital mortality related to COVID-19 (n= 58/905, 6.4%)						
Age at admission[1]	1.04	1.02-1.06	***<0.001***	1.05	1.03-1.07	**<0.001**
Male[2]	2.24	1.29-3.98	***0.005***	2.08	1.19-3.75	**0.01**
Immunocompromised[3]	2.56	1.46-4.42	***<0.001***	3.47	1.90-6.30	**<0.001**
Unvaccinated[4]	0.58	0.14-1.66	0.38	-	-	-
Boosted[5]	1.08	0.55-2.25	0.83	-	-	-
Viral vector vaccination[6]	1.47	0.64-3.22	0.34	-	-	-
Vaccinated <2 months before admission[7]	0.30	0.05-1.05	0.11	-	-	-
Vaccinated <4 months before admission[7]	1.09	0.44-3.08	0.87	-	-	-
Vaccinated <6 months before admission[7]	0.96	0.32-4.13	0.94	-	-	-

Supplemental Table S5. Mixed-model logistic regression analysis adult patients infected with Omicron comparing adult patients infected with Omicron and moderate/severe/critical/fatal COVID-19 (n=323) (*i.e.,* excluding asymptomatic patients or patients with mild COVID-19 symptoms). Covariates: 1. odds increase per year; 2. versus female; 3. versus non-immunocompromised; 4. versus any vaccination; 5. versus unvaccinated or (1/2) basic vaccination; 6. versus vaccinated with mRNA solely. 7. versus unvaccinated or vaccinated >2/>4/>6 months before admission. Abbreviations: OR, odds ratio; CI, confidence interval.

	**Crude OR**	**95% CI**	**p value**	**Adjusted OR**	**95% CI**	**p value**
**Outcome 1** – disease severity: odds of critical/fatal COVID-19 (n=92/323, 28.5%)						
Age at admission[1]	1.01	0.99-1.02	0.32	1.02	1.00-1.03	0.06
Male[2]	1.60	0.96-2.68	0.07	1.63	0.98-2.76	0.06
Immunocompromised[3]	2.12	1.24-3.58	***0.005***	2.42	1.39-4.22	***0.002***
Unvaccinated[4]	0.69	0.27-1.55	0.40	-	-	-
Boosted[5]	0.89	0.50-1.59	0.68	-	-	-
Viral vector vaccination[6]	0.85	0.44-1.60	0.62	-	-	-
Vaccinated <2 months before admission[7]	0.80	0.32-1.78	0.60	-	-	-
Vaccinated <4 months before admission[7]	0.80	0.41-1.58	0.51	-	-	-
Vaccinated <6 months before admission[7]	0.84	0.35-2.25	0.72	-	-	-
**Outcome 2** – ICU admission: odds of admission or transferred to ICU due to COVID-19 (n=68/323, 21.1%)						
Age at admission[1]	0.96	0.95-0.98	***<0.001***	0.97	0.95-0.98	***<0.001***
Male[2]	1.06	0.61-1.86	0.84	0.97	0.54-1.75	0.93
Immunocompromised[3]	1.79	0.95-3.35	0.07	1.96	1.06-3.57	***0.03***
Unvaccinated[4]	0.99	0.40-2.19	0.97	-	-	-
Boosted[5]	0.77	0.43-1.41	0.39	-	-	-
Viral vector vaccination[6]	0.97	0.50-1.85	0.94	-	-	-
Vaccinated <2 months before admission[7]	1.65	0.75-3.45	0.20	-	-	-
Vaccinated <4 months before admission[7]	0.83	0.43-1.67	0.59	-	-	-
Vaccinated <6 months before admission[7]	0.80	0.33-2.13	0.62	-	-	-
**Outcome 3** – mortality: odds of in-hospital mortality related to COVID-19 (n=58/323, 18.0%)						
Age at admission[1]	1.04	1.02-1.06	***0.001***	1.06	1.03-1.09	***<0.001***
Male[2]	1.42	0.78-2.63	0.25	1.63	0.87-3.13	0.13
Immunocompromised[3]	1.79	0.95-3.35	0.07	3.13	1.52-6.62	***0.002***
Unvaccinated[4]	0.56	0.13-1.66	*0.35*	-	-	-
Boosted[5]	0.92	0.44-1.98	*0.82*	-	-	-
Viral vector vaccination[6]	1.25	0.53-2.83	*0.60*	-	-	-
Vaccinated <2 months before admission[7]	0.44	0.07-1.59	*0.28*	-	-	-
Vaccinated <4 months before admission[7]	1.09	0.42-3.17	*0.87*	-	-	-
Vaccinated <6 months before admission[7]	0.84	0.26-3.77	*0.80*	-	-	-

Supplemental Table S6. Differences in total hospital length-of-stay (days) in adult patients infected with Omicron and moderate/severe/critical/fatal COVID-19 (n=323), dependent on COVID-19 symptoms and immune status or vaccination status at admission.

	**Hospital length-of-stay (days)**				
	**Worst COVID-19 symptoms during admission**				**All symptomatic patients N = 323**
	**Moderate N = 89**	**Severe N = 142**	**Critical N = 34**	**Fatal N = 58**	
**Immune status**					
Immunocompromised	5 (4-7) N = 29	8 (6-17) N = 30	42 (18-67) N = 16	18 (6-36) N = 23	9 (5-25) N = 184
Non-immunocompromised	10 (5-19) N = 60	8 (5-15) N = 112	17 (12-38) N = 18	11 (5-17) N = 35	10 (5-18) N = 657
p-value	***0.005***	0.96	0.18	0.09	0.75
**Vaccination status**					
Unvaccinated	7 (3-34) N= 11	8 (4-18) N = 19	39 (24-56) N = 4	7 (6-12) N = 3	8 (4-28) N = 37
Vaccinated	7 (4-14) N = 74	8 (6-16) N = 117	21 (12-59) N = 30	12 (5-26) N = 35	9 (5-18) N = 256
p-value	0.57	0.76	0.72	0.63	0.82
Unboosted	4 (2-17) N = 26	7 (4-15) N = 39	29 (8-63) N = 11	10 (5-14) N = 12	8 (4-17) N = 88
Boosted	8 (5-14) N = 59	8 (5-12) N = 97	22 (14-59) N = 23	13 (5-26) N = 26	10 (6-19) N = 205
p-value	0.08	0.08	0.77	0.71	***0.03***
Viral vector	8 (6-18) N = 22	9 (6-16) N = 40	35 (12-57) N = 7	16 (11-39) N = 10	10 (6-19) N = 79
mRNA	6 (4-13) N = 51	8 (6-14) N = 73	27 (21-77) N = 23	9 (3-27) N = 17	8 (4-17) N = 164
p-value	0.25	0.39	0.29	0.42	0.12

Supplemental Figure S1. Number of newly hospitalized patients with positive SARS-CoV-2 screening, divided by age, date of admission and VOC. A. Children (<18 years old); B. Adults (≥18 years old).

A

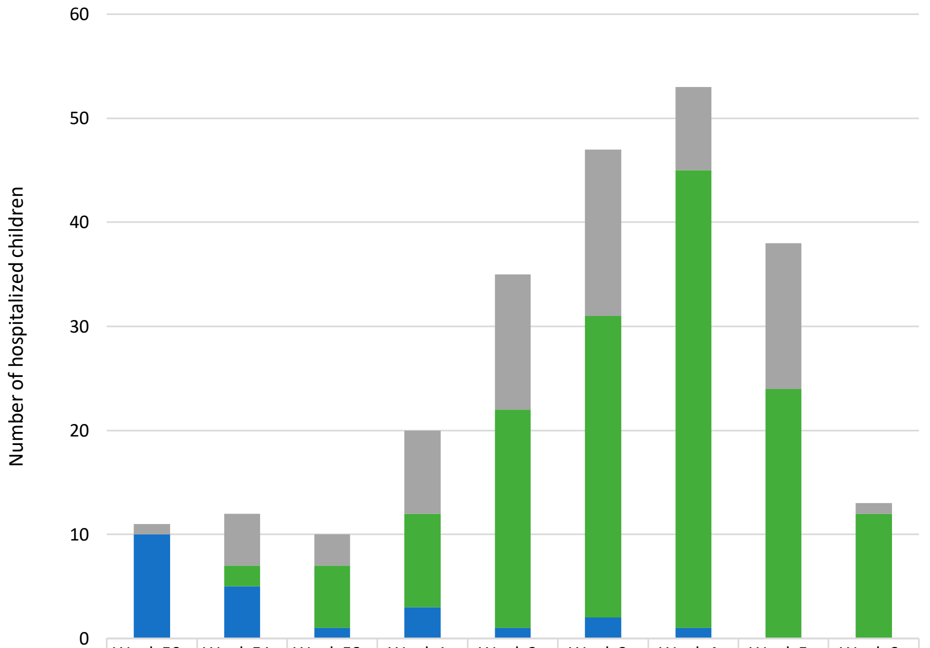

	Week 50 - 2021	Week 51 - 2021	Week 52 - 2021	Week 1 - 2022	Week 2 - 2022	Week 3 - 2022	Week 4 - 2022	Week 5 - 2022	Week 6 - 2022
Untyped	1	5	3	8	13	16	8	14	1
Omicron		2	6	9	21	29	44	24	12
Delta	10	5	1	3	1	2	1		

Date of admission (weeks)

B

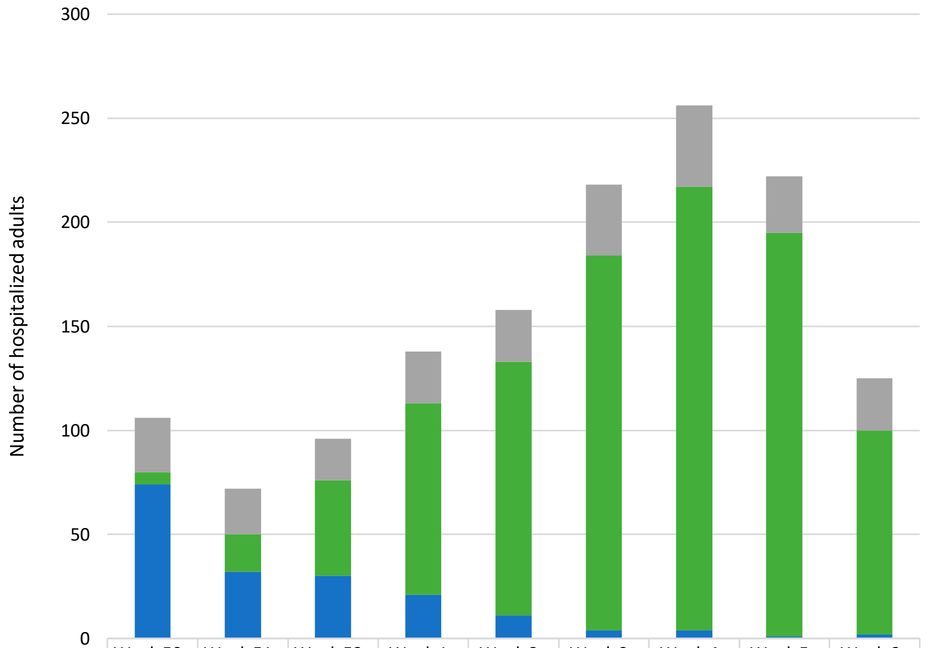

	Week 50 - 2021	Week 51 - 2021	Week 52 - 2021	Week 1 - 2022	Week 2 - 2022	Week 3 - 2022	Week 4 - 2022	Week 5 - 2022	Week 6 - 2022
Untyped	26	22	20	25	25	34	39	27	25
Omicron	6	18	46	92	122	180	213	194	98
Delta	74	32	30	21	11	4	4	1	2

Date of admission (weeks)

Supplemental Figure S2. Number of newly hospitalized patients with positive SARS-CoV-2 screening, divided by age and classification during hospitalization. A. Children (<18 years old); B. Adults (≥18 years old).

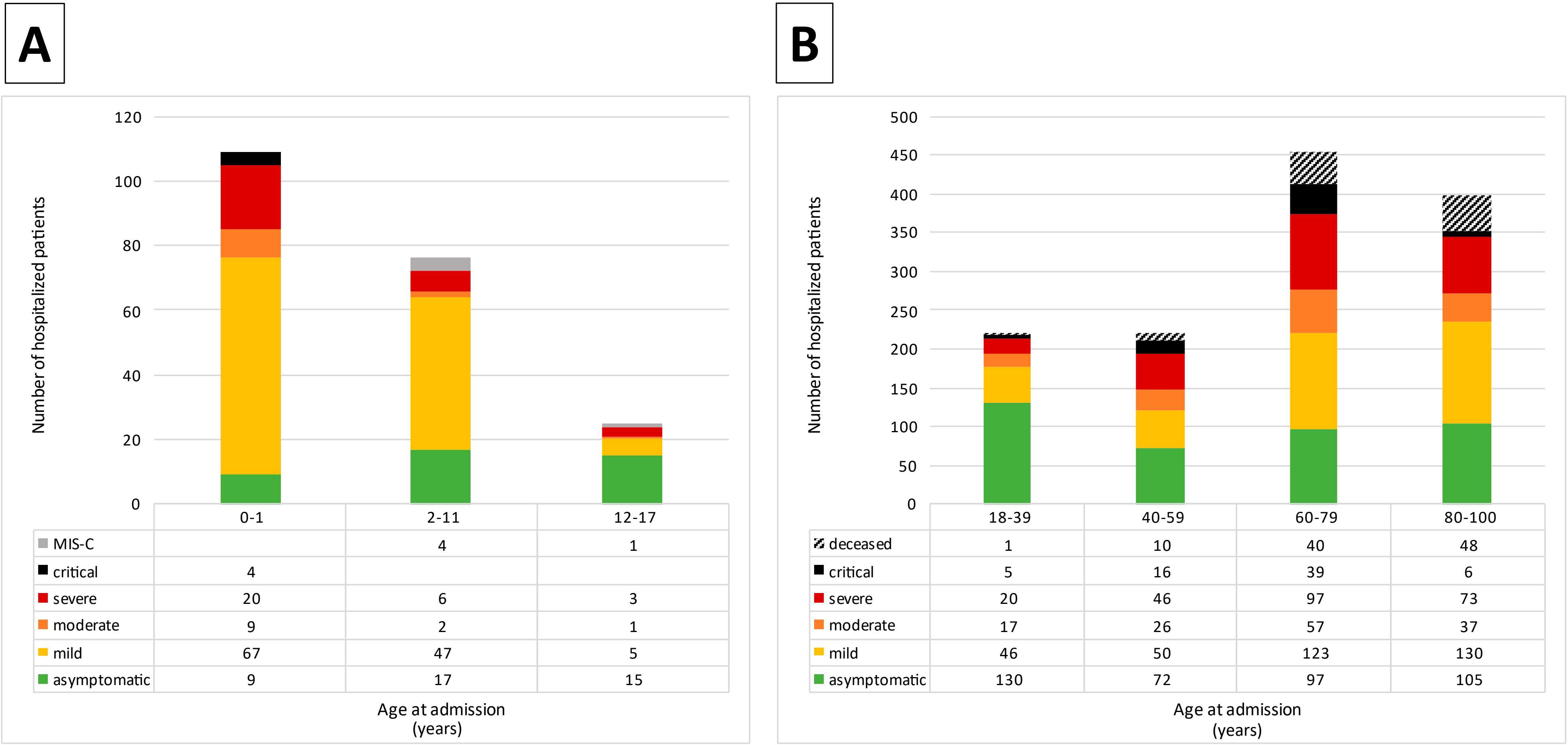

A

	0-1	2-11	12-17
MIS-C		4	1
critical	4		
severe	20	6	3
moderate	9	2	1
mild	67	47	5
asymptomatic	9	17	15

B

	18-39	40-59	60-79	80-100
deceased	1	10	40	48
critical	5	16	39	6
severe	20	46	97	73
moderate	17	26	57	37
mild	46	50	123	130
asymptomatic	130	72	97	105

Supplemental Materials & Methods

A. Classification COVID-19 symptoms

To classify patients based on COVID-19 symptoms, NIH guidelines [1] were used as symptom definition:

1) asymptomatic [or presymptomatic] *i.e.,* no symptoms that are consistent with COVID-19
2) mild illness *i.e.,* any of the various signs and symptoms of COVID-19 (e.g., fever, cough, sore throat, malaise, headache, muscle pain, nausea, vomiting, diarrhea, loss of taste and smell) but who do not have shortness of breath, dyspnea, or abnormal chest imaging
3) moderate illness *i.e.,* evidence of lower respiratory disease during clinical assessment or imaging and who have an oxygen saturation (SpO2) ≥94% on room air at sea level
4) severe illness *i.e.,* SpO2 <94% on room air at sea level, a ratio of arterial partial pressure of oxygen to fraction of inspired oxygen (PaO2/FiO2) <300 mm Hg, a respiratory rate >30 breaths/min, or lung infiltrates >50%. *Remark*: A patient can only be classified as "severe illness" if he/she also has clinical signs of lower respiratory tract infection (via clinical examination or via imaging = definition of "moderate illness"). So, individuals who have SpO2 <94% on room air at sea level but no evidence of lower respiratory disease are NOT classified as “severe illness”
5) critical illness *i.e.,* respiratory failure, septic shock, and/or multiple organ dysfunction. *Remark*: a patient who is admitted with septic shock with no respiratory clinic and who is clearly NOT linked to the (accidentally found) positive SARS-CoV-2 screening is NOT classified as "critical illness”
6) MIS-C *i.e.,* multisystem inflammatory syndrome in children associated with COVID-19 (case definition: CDC [2]).
7) fatal: deceased due to COVID-19. *Remark*: a patient who dies where the (incidentally found) positive SARS-CoV-2 screening is clearly unrelated to the death is not classified as "deceased due to COVID-19. However, from the slightest suspicion that COVID-19 may have contributed to the death of the patient, the patient is classified as “deceased due to COVID-19”

B. Classification immune status

To classify patients as immunocompromised, CDC criteria [3] for moderately or severely immunocompromised people are applied:

1) a patient receiving active cancer treatment for tumors or cancers of the blood
2) a patient who received an organ transplant and is taking medicine to suppress the immune system
3) a patient who received a stem cell transplant within the last 2 years or is taking medicine to suppress the immune system
4) a patient with moderate or severe primary immunodeficiency (such as DiGeorge syndrome, Wiskott-Aldrich syndrome – see categorization Inborn errors of immunity (primary immunodeficiencies), UpToDate 2022 [4])
5) a patient with advanced or untreated HIV infection
6) a patient with active treatment with high-dose corticosteroids (defined as a daily intake of ≥15mg of prednisolone or its equivalent for >2 weeks [5]) or other drugs that may suppress your immune response

[1] COVID-19 Treatment Guidelines Panel. Coronavirus Disease 2019 (COVID-19) Treatment Guidelines. National Institutes of Health. Available at https://www.covid19treatmentguidelines.nih.gov/overview/clinical-spectrum/. Accessed January 10, 2022

[2] CDC Health Alert Network. MIS-C associated with COVID-19. Available via https://emergency.cdc.gov/han/2020/han00432.asp. Accessed February 10, 2022

[3] CDC. COVID-19 Vaccines for Moderately or Severely Immunocompromised People. Available via https://www.cdc.gov/coronavirus/2019-ncov/vaccines/recommendations/immuno.html. Accessed February 9, 2022

[4] Sullivan KE, Jyonouchi SC. Inborn errors of immunity (primary immunodeficiencies): Classification (UpToDate, last updated December 15, 2021)). Available via https://www.uptodate.com/contents/inborn-errors-of-immunity-primary-immunodeficiencies-

[5] Shroff A, Mertz D. Infectious Diseases Risk Whole in Chronic, High-Dose Corticosteroids. Canadian Journal of General Internal Medicine;2017;12(1):10-13

C. Classification vaccination status

Vaccination status as registered at time of admission were registered, including date of last administrated vaccine. Abbrevations: Pfizer vaccine, BNT162b2 (COMIRNATY), Pfizer, Inc., and BioNTech [mRNA vaccine]; Moderna vaccine, mRNA-1273 (Spikevax), ModernaTX, Inc. [mRNA vaccine]; AstraZeneca vaccine, ChAdOx1-S [recombinant] (Vaxzevria), Oxford/AstraZeneca [viral vector vaccine]; J&J vaccine, JNJ-78436735, Janssen Pharmaceuticals Companies of Johnson & Johnson [viral vector vaccine]

1) Unvaccinated
2) 1x Pfizer vaccine (1/2 basic vaccination, mRNA vaccination)
3) 2x Pfizer vaccine (basic vaccination, mRNA vaccination)
4) 3x Pfizer vaccine (boosted, mRNA vaccination)
5) 2x Pfizer vaccine + 1x Moderna vaccine (boosted, mRNA vaccination)
6) 1x Moderna vaccine (1/2 basic vaccination, mRNA vaccination)
7) 2x Moderna vaccine (basic vaccination, mRNA vaccination)
8) 3x Moderna vaccine (boosted, mRNA vaccination)
9) 2x Moderna vaccine + 1x Pfizer vaccine (boosted, mRNA vacintion)
10) 1x AstraZeneca vaccine (1/2 basic vaccination, viral vector vaccination)
11) 2x AstraZeneca vaccine (basic vaccination, viral vector vaccination)
12) 2x AstraZeneca vaccine + 1x Pfizer vaccine (boosted, counted as viral vector vaccination)
13) 2x AstraZeneca vaccine + 1x Moderna vaccine (boosted, counted as viral vector vaccination)
14) 1x J&J vaccine (basic vaccination, viral vector vaccination)
15) 1x J&J vaccine + 1x Pfizer vaccine (boosted, counted as viral vector vaccination)
16) 1x J&J vaccine + 1x Moderna vaccine (boosted, counted as viral vector vaccination)
17) ½ basic vaccination, vaccines unknown
18) Basic vaccination, vaccines unknown
19) Boosted, vaccines unknown
20) Vaccination status unknown

D. Used PCR, WGS and serology analyzers and assay in the participating centers. Abbreviations: N.A., not applicable

**Laboratory**	**PCR analyses**	**WGS analyses**	**Marker PCR analyses**	**Serology analyses**
University Hospitals Leuven	Analyzer: Panther (Hologic); KingFisher + Quantstudio 7 flex (Thermofisher); Alinity m (Abbott); GeneXpert (Cepheid)<br>Assay: MVPII nuclear extraction followed by TaqPath COVID-19 RT PCR (Thermo Fisher); Aptima SARS-CoV-2 (Hologic); Alinity m SARS-CoV-2 (Abbott molecular); Xpert SARS-CoV-2 (Cepheid)	Analyzer: GridION (Oxford Nanopore Technologies)<br>Assay: COVID Midi kit (C19MIDI; Oxford Nanopore Technologies) / Artic nCoV-2019 last version	Analyzer: KingFisher + Quantstudio 5/7 flex (Thermofisher)<br>Assay: MVPII nuclear extraction followed by Taqman™ SARS-CoV-2 Mutation Panel Assay	Analyzer: Architect (Abbott)<br>Assay: SARS-CoV-2 IgG II Quant (anti-S) & SARS-CoV-2 IgG assay (anti-N)
University Hospitals Brussel	Analyzer: AltoStar® (Altona Diagnostics); GeneXpert (Cepheid)<br>Assay: Altostar® SARS-CoV-2 RT- kit 1.5 (Altona Diagnostics); Xpert SARS-CoV-2 (Cepheid)	Analyzer: GridION (Oxford Nanopore Technologies)<br>Assay: 1200bp amplicon “midnight” primer set, using Nanopore Rapid Barcoding Kit	N.A.	N.A.
ZNA Antwerp	Analyzer: Cobas 6800 (Roche)<br>Assay: RT-PCR	Analyzer: MiSeq (Illumina)<br>Assay: AmpliSeq SARS-CoV-2	Analyzer: KingFisher + Quantstudio 5 (Thermofisher)<br>Assay: MVPII nuclear extraction followed by TaqPath COVID-19 RT PCR (Thermo Fisher)	N.A.
OLV Hospital Aalst	Analyzer: Starlet & Biorad<br>Assay: In-house PCR (N1 & E-gene)	Analyzer: MinION (Oxford Nanopore Technologies)	Assay: In-house PCR assays (multiplex)	Analyzer: Cobas (Roche)
Laboratoire Hospitalier Universitaire de Bruxelles	Analyzer: Alinity m (Abbott); Abbott-M2000 (Abbott)<br>Assay: Alinity m SARS-CoV-2; Abbott Real Time SARS-CoV-2	Analyzer: N.A. (Oxford Nanopore Technologies)<br>Assay: Artic nCoV-2019 last version	N.A.	Analyzer: Liaison XL<br>Assay: Liaison SARS-CoV-2 Trimerics IgG
AZ Sint-Jan Brugge-Oostende	Analyzer: GeneXpert (Cepheid); Cobas 6800 (Roche)<br>Assay: Xpert SARS-CoV-2 (Cepheid); Cobas® SARS-CoV-2 (Roche)	Analyzer: MiSeq (Illumina)<br>Assay: QIAseq® DIRECT SARS-CoV-2	Analyzer: ViiA7<br>Assay: Taqman™ SARS-CoV-2 Mutation Panel Assay	Analyzer: Architect (Abbott)<br>Assay: SARS-CoV-2 IgG II Quant (anti-S) & SARS-CoV-2 IgG assay (anti-N)

Jessa Hospital Hasselt	Analyzers: Alinity M (Abbott), GeneXpert GX16 (Cepheid), Cobas LIAT System (Roche), Luminex Aries (Thermo Fisher), M2000 (Abbott)	Analyzer: MiSeq (Illumina)<br>Assay: AmpliSeq SARS-CoV-2	N.A.	Analyzer: Alinity I (Abbott)<br>Assay: SARS-CoV-2 IgG anti-S
University Hospitals Antwerp	Analyzer: BD MAX System (BD); GeneXpert (Cepheid)<br>Assay: SARS-CoV-2 for BD MAX (BD); Xpert SARS-CoV-2 (Cepheid)	Analyzer: NextSeq (Illumina)<br>Assay: COVIDSeq	Analyzer: KingFisher + Quantstudio 7 flex (Thermofisher)<br>Assay: MVPII nuclear extraction followed by TaqPath COVID-19 RT PCR (Thermo Fisher)	N.A.
